# Impact of protein prenylation inhibition on *Mycobacterium leprae* viability and IL-1β production in infected macrophages

**DOI:** 10.1128/jb.00185-25

**Published:** 2025-08-27

**Authors:** Matheus da Silva Rocha, Antônio Marcos Rodrigues Pereira, Plínio Marcos Freire dos Santos, André Alves Dias, Melissa Pontes Pereira, Patrícia Sammarco Rosa, Daniele F. F. Bertoluci, John T. Belisle, Fabricio da Mota Ramalho Costa, Cristiana Santos de Macedo, Maria Cristina Vidal Pessolani, Marcia Berrêdo-Pinho

**Affiliations:** 1 Laboratory of Cellular Microbiology, Oswaldo Cruz Institute, Oswaldo Cruz Foundation (FIOCRUZ) 196605 , Rio de Janeiro, Brazil; 2 Divisão de Pesquisa e Ensino, Instituto Lauro de Souza Lima, São Paulo, Brazil; 3 Department of Microbiology, Immunology and Pathology, Colorado State University, Fort Collins, Colorado, USA; 4 Center for Technological Development in Health (CDTS), Oswaldo Cruz Foundation (FIOCRUZ) 37903 , Rio de Janeiro, Brazil; University of Notre Dame, Notre Dame, Indiana, USA

**Keywords:** isoprenoids, IL-1β, inflammasome, prenylation, leprosy, mevalonate pathway

## Abstract

Leprosy is a chronic infectious disease caused by *Mycobacterium leprae* and *M. lepromatosis*. Brazil consistently ranks among the countries with the highest number of leprosy cases. Data from our group showed that *M. leprae* upregulates the mevalonate pathway (MP), contributing to the accumulation of cholesterol-ester-enriched lipid droplets in infected macrophages, and that the inhibition of this pathway by statins decreases bacterial intracellular viability. It has already been shown that part of the deleterious effect of statins on *M. leprae* survival is related to the reduced cholesterol levels, which *M. leprae* oxidizes to generate reductive power. According to the literature, statins, by inhibiting MP, increase the production of the inflammatory cytokine IL-1β through the negative modulation of the biosynthesis of the isoprenoid geranylgeranyl pyrophosphate (GGPP) and farnesyl pyrophosphate (FPP), which are responsible for protein prenylation. In the present study, we tested the hypothesis that part of the effect of statins on the intracellular viability of *M. leprae* comes from their impact on IL-1β production via decreased prenylation. We demonstrate that GGPP is essential in macrophage*–M. leprae* interaction since the MP inhibitors, pamidronate and GGTI-298, which inhibit the enzymes farnesyl pyrophosphate synthase and geranylgeranyl transferase-1, respectively, decreased the intracellular viability of *M. leprae*, measured by RT-qPCR. MP inhibitors increased IL-1β production by activating the inflammasome, but this effect was reversed with GGPP. IL-1β levels were inversely related to bacterial survival. In conclusion, our findings highlight the potential role of protein geranylgeranylation in *M. leprae* pathogenesis and suggest new therapy options for leprosy.

**IMPORTANCE:**

*Mycobacterium leprae*, the bacterium that causes leprosy, survives and replicates inside macrophages. Statins, which inhibit the mevalonate pathway, promote bacterial killing in macrophages by affecting cholesterol and isoprenoid production. Cholesterol is crucial for *M. leprae* survival in macrophages, which explains the microbicidal effect of statins on the bacteria. However, the role of isoprenoid inhibition in statin-induced bacterial killing has not been explored. Isoprenoid groups are added to about 2% of the mammalian proteins, ensuring their proper function. This study focused on geranylgeranyl pyrophosphate (GGPP) and found that inhibiting GGPP formation or protein prenylation in infected macrophages triggered IL-1β production, thereby controlling mycobacterial infection. The findings highlight the importance of protein prenylation in *M. leprae* and suggest new therapeutic strategies for leprosy.

## INTRODUCTION

Protein prenylation is a crucial post-translational modification that allows proteins to associate with cellular membranes, establishing specific localization necessary for their function (1). This process involves adding one or more isoprenoid groups, such as geranylgeranyl or farnesyl pyrophosphates (GGPP or FPP, respectively), impacting approximately 2% of the mammalian proteome (2, 3). Prenylation is facilitated by three key prenyltransferases: geranylgeranyl transferase-1 (GGTase-1), which is primarily involved in the prenylation of small GTPases, such as Rac1, Rac2, RhoA, RhoB, and RalA; geranylgeranyl transferase-2 (GGTase-2), responsible for modifying most Rab family proteins; and farnesyl transferase (FTase), which primarily acts on the Ras superfamily small GTPases (4). Most prenylated proteins are CAAX proteins; the “CAAX” box consists of a C-terminal signal sequence: “C” denotes a cysteine residue, “AA” indicates two aliphatic residues, and “X” represents any C-terminal amino acid, which vary according to substrate specificity for different transferases (5–7). Disruptions in protein prenylation have been linked to various disorders, and recent studies suggest that protein prenylation also influences cytokine production and modulates immune responses (8–10).

Patients with mevalonate kinase deficiency (MKD) exhibit increased levels of the pro-inflammatory cytokine IL-1β, which correlates with inflammasome activation and caspase-1 activity (10, 11). This increase arises from reduced prenylation of specific GTPases due to a lack of isoprenoids, resulting from metabolic pathway disturbances associated with the deficiency (10, 11). Furthermore, drugs targeting the cholesterol biosynthetic pathway have been shown to modulate immune responses (10, 12, 13). By inhibiting isoprenoid synthesis, these drugs reduce the prenylation of key GTPases, such as Rac1 and RhoA, leading to increased GTPase activity and subsequent inflammasome activation (10, 11, 14). This activation ultimately releases the pro-inflammatory cytokines IL-1β and IL-18, which are implicated in various inflammatory diseases (10, 11, 15). IL-1β is an important pro-inflammatory cytokine that plays a crucial role in the early immune response. It initiates the inflammatory process and aids the subsequent adaptive immune response by participating in signaling pathways across various cell types (16, 17). IL-1β plays a dual role in leprosy, contributing both to mounting an antimicrobial response against *M. leprae* and to the acute inflammatory response and tissue damage observed in leprosy reactional episodes (18).

The obligate intracellular pathogen *Mycobacterium leprae*, the etiological agent of leprosy, primarily infects skin macrophages and Schwann cells of peripheral nerves (19). If left untreated, leprosy can lead to progressive and permanent disabilities (19). However, *M. lepromatosis*, a bacterium closely related phylogenetically to *M. leprae*, has recently been identified as the causative agent of diffuse lepromatous leprosy associated with Lucio’s phenomenon (20, 21). Since 1981, the World Health Organization (WHO) has stated that all patients with leprosy should be treated with multidrug therapy (MDT) for a period of 6 months to 1 year, and even though leprosy is treatable, it continues to be a significant public health challenge in many resource-limited countries, such as India and Brazil (22). In 2021, the WHO established the "Towards Zero Leprosy: Global Leprosy (Hansen’s disease) Strategy 2021–2030" (23), which aims to eliminate leprosy as a public health issue, and one of the key strategies for achieving this goal is the development of new drugs that can complement multidrug therapy (MDT), addressing the challenges posed by drug-resistant strains, lengthy treatments, and drug-related side effects that can impact patient adherence and quality of life (24).

The mevalonate pathway (MP) is one of the host cell pathways modulated by *M. leprae* in infected macrophages (25). As previously demonstrated by our group, upon infection, *M. leprae* induces the formation of lipid droplets (LDs) enriched in cholesterol by upregulating the MP and promoting the uptake of exogenous cholesterol (25). Of note, cholesterol colocalizes to *M. leprae-*containing phagosomes, and inhibition of 3-hydroxy-3-methylglutaryl-CoA (HMG-CoA reductase), the rate-limiting enzyme for the biosynthesis of cholesterol, by statins reduces intracellular cholesterol levels and LD accumulation with a concomitant decrease in bacillary viability in both *in vitro* and *in vivo* studies (25, 26). The direct link between intracellular cholesterol availability and bacterial survival can, at least in part, be explained by the capacity of *M. leprae* to uptake and oxidize host cell cholesterol to cholestenone, generating reductive power for ATP and mycobacterial lipid synthesis (27, 28). However, the possibility that statins’ killing effect on *M. leprae* is also related to their capacity to inhibit the biosynthesis of isoprenoids, which, as mentioned above, activates the innate immune response, cannot be excluded.

In the present study, we investigated the potential effects of MP blockage on protein prenylation inhibition in THP-1 macrophages. We found that *M. leprae-*stimulated cells showed increased release of IL-1β and caspase-1 activation with concomitant loss of bacterium viability when prenylation is inhibited. In addition, we found that this effect is associated with decreased production of GGPP. This discovery opens new possibilities for targeted interventions in leprosy.

## MATERIALS AND METHODS

### Mycobacteria

The *M. leprae* Thai-53 strain used was isolated from the hind footpads of athymic nu/nu mice, kindly provided by Dr. Patrícia Sammarco Rosa and Dr. Daniele Bertoluci at Lauro de Souza Lima Institute (Bauru-SP, Brazil). Briefly, about 9 months after inoculation of the bacilli (with a bacillary load of about 10^9^–10^10^ /g of tissue), the mice were euthanized, and *M. leprae* was purified from their footpads, as described in reference 29. The viability of the bacilli was assessed by fluorescence microscopy using the LIVE/DEAD BacLight Bacterial Viability Kit, following the manufacturer’s instructions. Once confirmed, the live *M. leprae* were quantified and used exclusively in the *in vitro* experiments of RT-qPCR for *M. leprae* viability (Fig. 9A and C). In contrast, for all other experiments, bacilli were inactivated by ionizing radiation (10 kGy) using a Gammacell 220 irradiator with a cobalt-60 source at the Nuclear Instrumentation Laboratory of the COPPE/Federal University of Rio de Janeiro (Rio de Janeiro, Brazil). All experiments were performed using either live or irradiated *M. leprae* at a multiplicity of infection (MOI) of 10:1.

### Cultivation of THP-1 macrophages

Human monocytic cell line THP-1 from ATCC no. TIB 202 was grown as a suspension in RPMI 1640 medium (Gibco) supplemented with 10% fetal bovine serum (FBS, Sigma-Aldrich) at 37°C and 5% CO2. The monocytes were plated and incubated for differentiation into macrophages with 50 ng/mL of PMA (phorbol-12-myristate-13-acetate, Sigma-Aldrich) for 24 hours at 37°C and 5% CO2. After that, the medium was discarded, and the non-adherent cells were removed and washed with phosphate-buffered saline (PBS, Sigma-Aldrich). Then, a fresh PMA-free RPMI with 10% FBS was added. The macrophages remained in the culture for 24 hours at 37°C and 5% CO2. After this period, the cells were either stimulated or infected with irradiated *M. leprae* or live *M. leprae,* respectively, and treated or not with different compounds as described below.

### Enzyme-linked immunosorbent assay (ELISA)

THP-1 macrophages were stimulated or not with irradiated *M. leprae* and treated or not with atorvastatin (2 µM), pamidronate (2.5 µM, 5 µM, and 10 µM), GGPP (2 µM, 5 µm, and 10 µM), GGTi-298 (1 µM, 3 µM, and 10 µM), and mevalonate (150 µM and 300 µM) (all purchased from Sigma-Aldrich), and maintained at 37°C with 5% CO2 for 24 hours. After that, the supernatants were collected and stored at −20°C until the assay was over. IL-1β and IL-18 were quantified by ELISA using a human IL-1 beta/IL-1F2 Quantikine ELISA kit (R&D Systems) or a human total IL-18/IL-1F4 Quantikine ELISA Kit (R&D Systems) according to the manufacturer’s instructions.

### Quantification of active caspase-1

THP-1 macrophages in 96-well plates were primed or not with lipopolysaccharide (LPS, Sigma-Aldrich, 100 ng/mL) for 4 hours, followed by 20 µM nigericin for 60 minutes (positive control), 10 µM pamidronate (24 hours), and stimulated or not with irradiated *M. leprae* at a bacterium:cell ratio (MOI) of 10:1, and maintained at 37°C and 5% CO2 for 24 hours. Active caspase-1 was detected in THP-1 macrophages through fluorescence analysis by a plate reader (Molecular Devices Gemini XS 96-well) using a commercial kit (FAM-FLICA Caspase Assay cat. #98, Immunochemistry Technologies), following the manufacturer’s protocol.

### RT-qPCR for *M. leprae* viability analysis

THP-1 macrophages were infected with live *M. leprae* (MOI 10:1) at 33°C for 24 hours in the presence or absence of GGPP (2 µM), methanol, GGPP’s vehicle, pamidronate (10 µM), or GGTi-298 (10 µM). After that, the mRNA was isolated using TRIzol (Sigma-Aldrich), according to the manufacturer’s instructions. Its integrity was assessed by electrophoresis on a 1.2% agarose gel, and samples that did not present any signs of degradation were considered for subsequent analysis. The RNA reverse transcription was performed using GoScript Master Mix according to the manufacturer’s instructions; however, before that, the DNA was removed from the RNA samples using a TURBO DNA-free kit (Thermo Fisher). The levels of 16S rRNA were measured relative to 16S DNA. For this, the LinRegPCR software was used to calculate the PCR efficiency for each biological experiment, as described 30. To normalize, we considered the efficiency corrections. The primers for the rRNA 16S were used for both cDNA and DNA: sense 5′-GAA ACT GCG AAT GGC TCA TTA AAT CA-3′ and antisense 5′- CCC GTC GGC ATG TAT TAG CTC T-3′. TaqMan real-time PCR assay (TaqMan probe 16S 5′-TGG TTC CTT TGG TCG CTC GCT CC-3′) was used for both cDNA and DNA, and *M. leprae* viability was determined by the comparative Ct method described by Martinez et al. (30). The viability was calculated as described in reference 31. Viia7 real-time PCR system and software (Thermo Fisher) were used, and the results are expressed as bacterial viability (%).

### Cellular toxicity

To evaluate drug toxicity in THP-1 macrophages in response to the compounds used in this study, increasing concentrations of both GGPP (0.5 µM, 1 µM, 2 µM, 5 µM, and 10 µM) and pamidronate (1 µM, 10 µM, 100 µM, and 1M) were employed. For this, THP-1 macrophages were plated into 96-well plates (10^4^ cells/well) and treated or not with GGPP and pamidronate in the concentrations described above for 24 hours, at 37°C and 5% CO_2_. DMSO 10% was used as a cell death control. After 24 hours, 3-(4,5-dimethylthiazol-2-yl)-2,5-diphenyltetrazolium bromide (MTT, Thermo Fisher) was added, and the formation of formazan was measured at 600 nm by spectrophotometry, as described in reference 32. Inhibitor concentrations were selected based on the dose-response experiments and literature reports to ensure effectiveness without inducing cytotoxicity.

### Statistical analysis

The arithmetic means and standard errors of the mean (SEM) for the test results were calculated using GraphPad Prism 9. To assess statistically significant differences among values, we used a one-way analysis of variance (ANOVA), followed by the Bonferroni *post hoc* test for multiple comparisons. Additionally, where applicable, a Student’s *t*-test was used to compare two conditions. A *P* value < 0.05 (*), 0.01 (**), 0.001 (***), or 0.0001 (****) was considered significant.

## RESULTS

### Atorvastatin induced the production of IL-1β in *M. leprae-*stimulated macrophages

**Fig 1:**
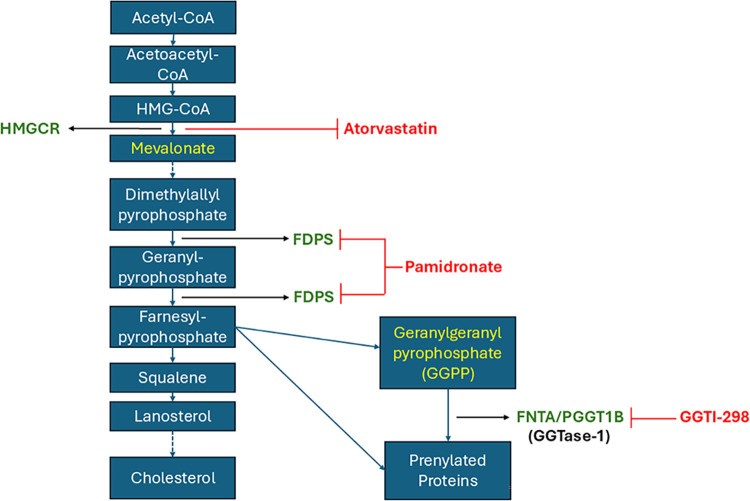
Mevalonate pathway diagram. Blue arrows indicate the main pathway steps, and red arrows indicate the points of action of the inhibitors used in this study: (1) atorvastatin, (2) pamidronate, and (3) GGTi-298. HMGCR: 3-hydroxy-3-methylglutaryl coenzyme A reductase; FDPS: farnesyl diphosphate synthase; FNTA: farnesyl transferase; CAAX box, subunit alpha; PGGT1B: protein geranylgeranyl transferase type I subunit beta.

Previous studies have shown that statins can enhance IL-1β production by macrophages (33–35). Therefore, we first investigated whether *M. leprae*-stimulated macrophages would behave in a similar way to non-stimulated cells, producing higher levels of IL-1β in response to statins, leading us to hypothesize that statins might reduce the viability of *M. leprae* not only by lowering cholesterol levels but also by activating the innate immune response. To test this hypothesis, we separately stimulated THP-1 macrophages with irradiated *M. leprae* treated with three different inhibitors. The use of irradiated *M. leprae* in macrophage studies is a well-established approach, primarily due to its ability to effectively mimic early events in host cell–pathogen interaction observed with live bacteria (36). Furthermore, macrophage lipid metabolism studies have shown that irradiated *M. leprae* can induce lipid droplet formation in macrophages, similarly to live bacilli (37). Therefore, all assays, except for the bacilli viability assays (Fig. 9A and C), were performed using irradiated *M. leprae*, due to the inherent challenges associated with obtaining sufficient live bacteria. Figure 1 presents a diagram illustrating the key steps of the MP, highlighting the inhibitors used in this study and the specific enzymes they target, such as atorvastatin, a HMG-CoA reductase inhibitor (HMGCR), pamidronate, inhibitor of farnesyl diphosphate synthase (FDPS), and GGTI-298, inhibitor of geranylgeranyl transferase type 1 (GGTase-1) (Fig. 1).

**Fig 2:**
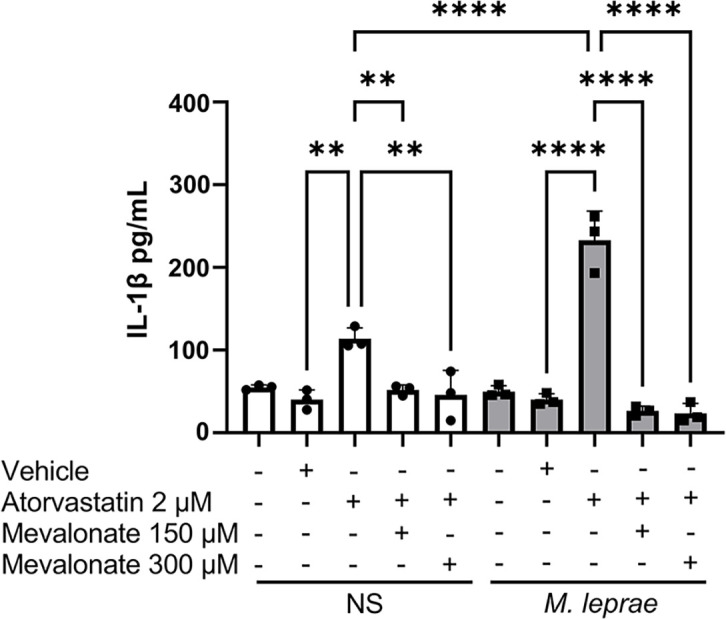
IL-1β modulation by atorvastatin in *M. leprae-*stimulated macrophages. THP-1 macrophages were stimulated or not with irradiated *M. leprae* at a bacterium:cell ratio of 10:1 and treated or not with atorvastatin (2 µM), mevalonate (150 µM and 300 µM), and atorvastatin vehicle (ethanol) for 24 hours to evaluate IL-1β production by ELISA. All data are presented as mean ± SD of three independent experiments run in duplicate. Statistical significance was determined using a one-way ANOVA followed by a post hoc test. **P < 0.01; ****P < 0.0001.

The results show that atorvastatin increased IL-1β production by about 107.3% in macrophages not stimulated with *M. leprae* (NS), compared to the control condition, i.e., not treated with atorvastatin (*P* = 0.0015) (Fig. 2). Regarding the *M. leprae-*stimulated macrophages, the presence of *M. leprae* could not induce IL-1β release above the background levels observed in NS cells (Fig. 2). However, the atorvastatin effect on *M. leprae-*stimulated cells was even more pronounced, increasing the production of IL-1β by 366.6% (*P* = 0.0009), when compared to *M. leprae-*stimulated cells, but not treated with atorvastatin (Fig. 2). To confirm that the effect of atorvastatin on IL-1β release was due to the inhibition of HMG-CoA reductase, mevalonate (150 µM or 300 µM), the product of the enzyme, was added to the culture medium. Mevalonate, at both concentrations, reversed the atorvastatin-induced effect to the control level in both conditions (Fig. 2).

### The isoprenoid GGPP abolished IL-1β upregulation induced by atorvastatin

**Fig 3:**
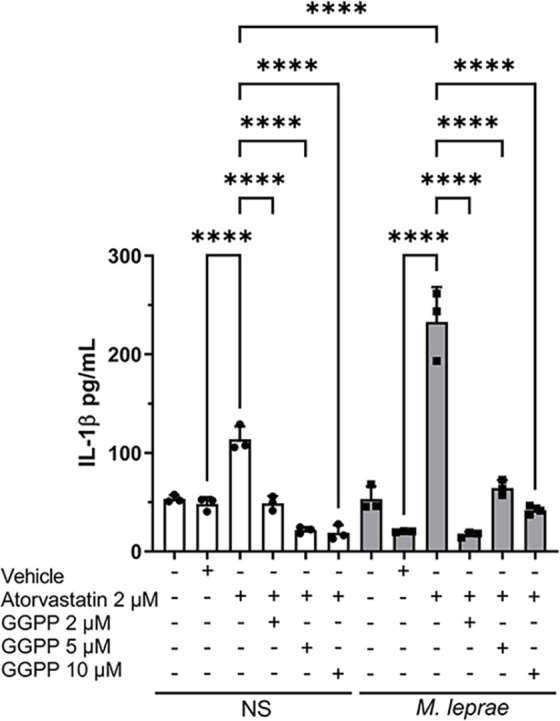
IL-1β induction by atorvastatin is reversed by GGPP. THP-1 macrophages were stimulated or not with *M. leprae* at a bacterium:cell ratio of 10:1 and treated or not with atorvastatin (2 µM), geranylgeranyl pyrophosphate (GGPP) (2 µM, 5 µM, or 10 µM), and GGPP vehicle (methanol) for 24 hours to evaluate IL-1β production by ELISA. All data are presented as mean ± SD of three independent experiments run in duplicate. Statistical significance was determined using a one-way ANOVA followed by a post hoc test. ****P < 0.0001.

The literature suggests that a deficiency in geranylgeranylated proteins can significantly increase IL-1β production in MKD (38, 39). We then tested whether the effect of atorvastatin on IL-1β production observed in *M. leprae*-stimulated THP-1 macrophages was linked to the reduction of GGPP, a MP downstream product involved explicitly in protein geranylation (Fig. 3). The addition of GGPP in all concentrations (2 µM, 5 µM, or 10 µM) reversed the effect on IL-1β production induced by atorvastatin in both NS and *M. leprae-*stimulated conditions (Fig. 3). It is important to note that GGPP was not cytotoxic in any of the evaluated concentrations (Fig. S1). These results suggest that *M. leprae*-stimulated macrophages behave similarly to control cells, with atorvastatin-induced IL-1β linked to low GGPP production and potentially reduced protein geranylgeranylation.

### FPPS inhibition induced IL-1β production in *M. leprae-*stimulated macrophages

**Fig 4:**
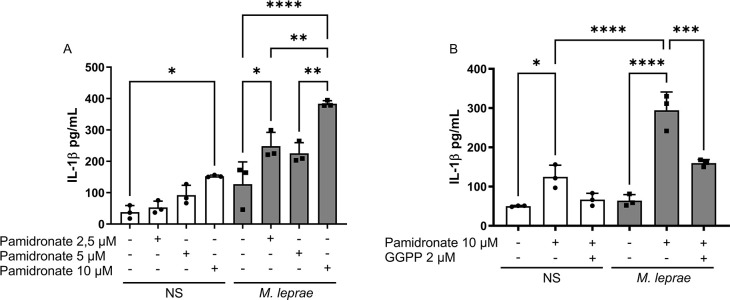
Direct isoprenoid synthesis inhibition increased IL-1β release by *M. leprae-*stimulated macrophages. THP-1 macrophages were stimulated or not with *M. leprae* at a bacterium:cell ratio of 10:1 and treated or not with (**A**) pamidronate (2.5 µM, 5 µM, or 10 µM) or (**B**) pamidronate (10 µM) and treated with geranylgeranyl pyrophosphate (2 µM) or not for 24 hours to evaluate IL-1β production by ELISA. All data are presented as mean ± SD of three independent experiments run in duplicate. Statistical significance was determined using a one-way ANOVA followed by a post hoc test. *P < 0.05; **P < 0.01; ***P < 0.001, ****P < 0.0001.

Similar experiments were conducted with pamidronate to investigate the involvement of prenylation in IL-1β production by inhibiting another step of the MP, closer to isoprenoid synthesis. To do so, we used pamidronate, a nitrogen-containing bisphosphonate that acts downstream of the MP, specifically blocking farnesyl pyrophosphate synthase (FPPS) (40, 41) (Fig. 4A and B). Firstly, we performed a concentration curve of pamidronate, ranging from 2.5 to 10 µM. *M. leprae-*stimulated cells treated with 10 µM produced the highest levels of IL-1β, with a significant increase of 201.33% compared to the non-treated control (*P* = 0.0034) (Fig. 4A). No cytotoxic effect was observed at any pamidronate concentration (Fig. S1b), and 10 mM was chosen for subsequent experiments. Next, we evaluated whether the isoprenoid GGPP would similarly reduce the effect of pamidronate on IL-1β production, as observed with atorvastatin treatment. As expected, in both *M. leprae-*stimulated and NS macrophages, GGPP treatment (2 µM) reduced pamidronate’s effect on IL-1β production by 78.26% and 46.4% (*P* = 0.008 and *P* = 0.0407, respectively), suggesting that GGPP or protein geranylgeranylation is essential for the effect of these two MP inhibitors (Fig. 4B).

### Neither pamidronate nor atorvastatin affected the production of IL-6 and IL-8

**Fig 5:**
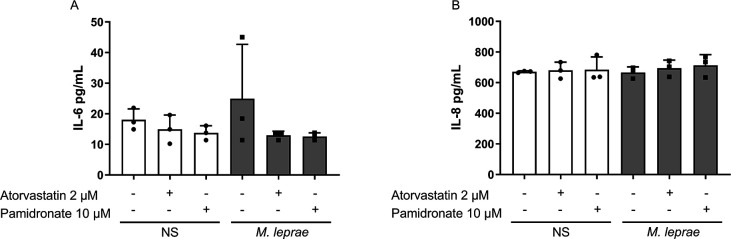
Analysis of IL-6 and IL-8 production upon MP blockage. THP-1 macrophages were stimulated or not with *M. leprae* at a bacterium:cell ratio of 10:1 and treated or not with atorvastatin (2 µM) or pamidronate (10 µM) for 24 hours to evaluate (A) IL-6 or (B) IL-8 production by ELISA. All data are presented as mean ± SD of three independent experiments run in duplicate. Statistical significance was determined using a one-way ANOVA followed by a post hoc test.

To determine whether pamidronate and atorvastatin treatment could induce the production of other pro-inflammatory cytokines, we measured IL-6 and IL-8 production (Fig. 5A and B). As shown in Fig. 5A and B, atorvastatin (2 µM) and pamidronate (10 µM) did not induce significant modulation in the production of these cytokines in either NS THP-1 macrophages or *M. leprae-*stimulated macrophages.

### Pamidronate induced caspase-1 activity in *M. leprae-*stimulated macrophages

**Fig 6:**
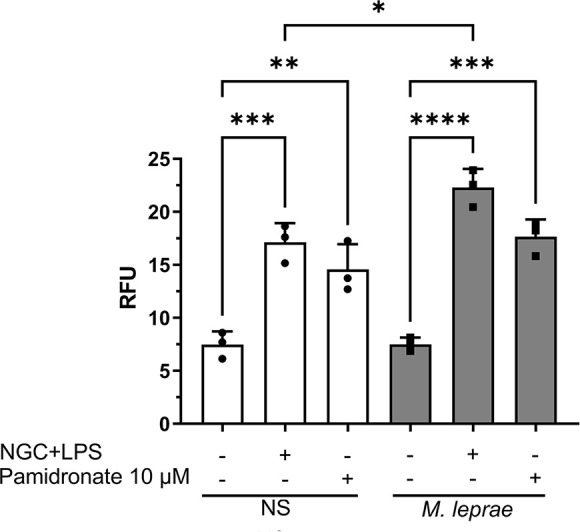
Pamidronate induced caspase-1 activity in *M. leprae-*stimulated macrophages: THP-1 macrophages were stimulated or not with *M. leprae* at a bacterium: cell ratio of 10:1 and treated or not with LPS (100 ng/mL) and nigericin (NGC, 20 µM), or pamidronate (10 µM) for 24 hours to evaluate caspase-1 activation. (Y-axis: relative fluorescence units [RFU]). All data are presented as mean ± SD of three independent experiments run in duplicate. Statistical significance was determined using a one-way ANOVA followed by a post hoc test. *P < 0.05; **P < 0.01; ***P < 0.001, ****P < 0.0001.

Blockage of the MP has been shown to induce inflammasome activation and IL-1β release due to impaired prenylation of specific geranylgeranylated GTPases, particularly of the Rho subfamily, resulting from a decreased availability of GGPP (10, 11). Next, we investigated inflammasome activation in THP-1 macrophages stimulated with *M. leprae* following pamidronate treatment. We used the FAM-FLICA (YVAD) kit to detect caspase-1 activity (Fig. 6). Pamidronate treatment (10 µM) induced caspase-1 activity in both NS and *M. leprae-*stimulated THP-1 macrophages, showing a significant increase of 94.8% and 135.9% of caspase-1 activity, respectively (*P* = 0.0102 and *P* = 0.0005). Similar caspase-1 activation was observed with LPS (100 ng/mL) followed by nigericin (NGC 20 µM), which was included as a positive control. The results are shown as relative fluorescence units (RFU), where higher RFU values indicate increased fluorescence intensity and, consequently, a greater level of the measured biological activity (in this case, caspase-1 activation). RFU is an arbitrary unit used to quantify fluorescence intensity, where the value is proportional to the amount of the fluorescently labeled product generated in the assay.

### Geranylgeranyl transferase-1 inhibition increased IL-1β and IL-18 production

**Fig 7:**
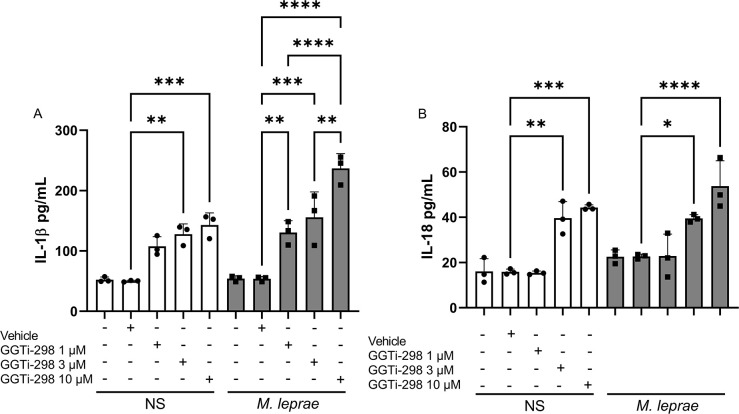
Specific geranylgeranyl transferase inhibition induced IL-1β and IL-18 production in *M. leprae-*stimulated macrophages. THP-1 macrophages were stimulated or not with *M. leprae* at a bacterium:cell ratio of 10:1 and treated or not with GGTi-298 (1 µM, 3 µM, or 10 µM) or vehicle (methanol) for 24 hours to evaluate IL-1β (**A**) and IL-18 (**B**) production by ELISA. All data are presented as mean ± SD of three independent experiments run in duplicate. Statistical significance was determined using a one-way ANOVA followed by a post hoc test. *P < 0.05; **P < 0.01; ***P < 0.001, ****P < 0.0001.

GGPP is a substrate for GGTase-1, an enzyme that catalyzes the geranylgeranylation of small GTPases, such as Rac1. Deficient prenylation of Rac1 increases its activation, promoting the conversion of pro-caspase 1 to active caspase 1 and inflammasome activation (10, 11). In this part of our investigation, we used the specific “CAAZ” peptidomimetic GGTi-298, a potent inhibitor of GGTase-1, to analyze its effect on IL-1β and IL-18 production—cytokines generated through the inflammasome pathway (42, 43)—by *M. leprae-*stimulated macrophages. Firstly, we performed a dose-response curve of GGTi-298 (1 µM, 3 µM, and 10 µM) (Fig. 7A and B). We observed that the conditions treated with 1, 3, or 10 µM of GGTi-298 in NS macrophages were able to significantly induce IL-1β, with 114.66%, 155.6%, and 185.9% , respectively (*P* = 0.0035, *P* = 0.0013, and *P* = 0.0013) (Fig. 7A). In *M. leprae-*stimulated macrophages, we also observed a significant induction with 1 µM, 3 µM, and 10 µM, but a higher induction was detected with 10 µM of GGTi-298, approximately 337.2% (*P* = 0.0002). To further support our hypothesis that the inhibition of geranylgeranylation contributes to inflammasome activation, we also evaluated whether GGTase-1 inhibition could modulate IL-18 production, another pro-inflammatory cytokine that is a well-established downstream effector of inflammasome activation (44). A similar result was observed with IL-18, showing significant induction in NS macrophages treated with GGTI-298 at 3 µM and 10 µM, with increases of 149.7% and 179.2% (*P* = 0.005 and *P* = 0.0001, respectively) (Fig. 7B). Also, we detected a significant induction in *M. leprae-*stimulated macrophages treated with 3 µM, showing 73.8% (*P* = 0.0001) (Fig. 7B), and a higher induction with 10 µM, showing 136.9% (*P* = 0.0088), similar to what we saw previously with IL-1β. No cytotoxic effect was observed at any tested GGTi-298 concentration (Fig. S2). These results indicate that protein geranylgeranylation also plays a significant role in the induction of IL-1β and IL-18 observed with MP inhibitors in *M. leprae-*stimulated macrophages.

### The increase in IL-1β production induced by GGTase-1 inhibition is not related to a reduction in cholesterol levels

**Fig 8:**
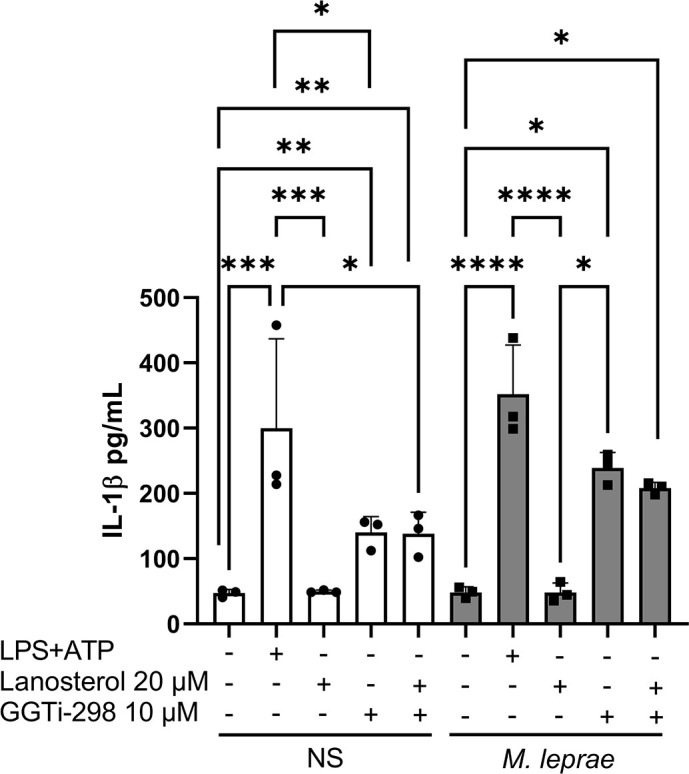
Lanosterol did not alter IL-1β production triggered by geranylgeranylation inhibition. THP-1 macrophages were stimulated or not with *M. leprae* at a bacterium:cell ratio of 10:1 and treated or not with LPS (100 ng/mL) for 4 hours followed by ATP (5 mM) for 30 minutes (positive control), lanosterol (20 µM), or GGTi-298 (10 µM) for 24 hours to evaluate IL-1β production by ELISA. All data are presented as mean ± SD of three independent experiments run in duplicate. Statistical significance was determined using a one-way ANOVA followed by a post hoc test. *P < 0.05; **P < 0.01; ***P < 0.001, ****P < 0.0001.

It has been described that the accumulation of GGPP, the substrate for GGTase-1, can inhibit HMG-CoA reductase activity through feedback regulation. Thus, to confirm that the increase in IL-1β production was related to the inhibition of geranylgeranylation and not to reduced cholesterol synthesis, *M. leprae-*stimulated THP-1 macrophages were treated with lanosterol (20 µM), a downstream intermediate of the MP and precursor of cholesterol (as illustrated in Fig. 1). As shown in Fig. 8, lanosterol supplementation did not affect IL-1β levels, either alone or in combination with the GGTase-1 inhibitor GGTI-298 (10 µM). Importantly, the addition of lanosterol failed to reverse the effect of GGTI-298. A positive control (LPS + ATP) confirmed the system was responsive by strongly inducing IL-1β. These findings reinforce that the pro-inflammatory response triggered by GGTase-1 inhibition is independent of cholesterol depletion and likely involves impaired protein prenylation.

### Inhibition of prenylation impaired *M. leprae* survival within infected macrophages, correlating with increased IL-1β production

**Fig 9:**
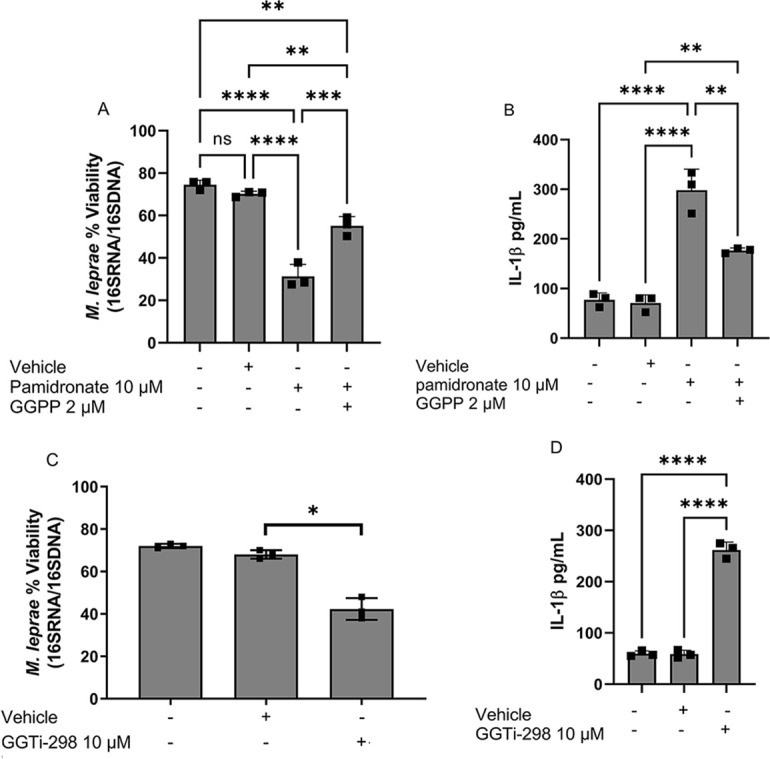
Inverse correlation between *M. leprae* intracellular viability and IL-1β production. THP-1 macrophages were infected with *M. leprae* at a 10:1 MOI and treated or not with pamidronate (10 µM), GGPP (2 µM), or GGTi-298 (10 µM) for 24 hours at 35°C and 5%CO_2_ to evaluate *M. leprae* viability, measured by RT-qPCR using the ratio of 16S cDNA/16S DNA (**A and C**) and IL-1β production by ELISA (**B and D**). All data are presented as mean ± SD of three independent experiments run in duplicate. Statistical significance was determined using a one-way ANOVA followed by a post hoc test. *P < 0.05; **P < 0.01; ***P < 0.001, ****P < 0.0001.

To test the hypothesis that part of the effect of MP inhibitors on the intracellular viability of *M. leprae* comes from its impact on IL-1β production via decreased protein prenylation, we added pamidronate and GGPP, or GGTi-298, to the culture medium of infected THP-1 macrophages. We used RT-qPCR to assess *M. leprae* viability and ELISA to quantify IL-1β production in these supernatants. Pamidronate treatment (10 µM) significantly reduced *M. leprae* viability, resulting in a 58.02% decrease (*P* = 0.0003) (Fig. 9A). However, concomitant treatment with GGPP (2 µM) significantly rescued bacterial viability back by 23.79% (*P* = 0.0047) (Fig. 9A). Analysis of IL-1β production in supernatants from infected THP-1 showed a 320.73% increase (*P* = 0.001) compared to the untreated infected control, and a subsequent 40.76% decrease (*P* = 0.0079) when cells were treated with both pamidronate and GGGP, compared to the condition treated only with pamidronate (Fig. 9B). This result suggests a correlation between increased IL-1β production and reduced bacterial viability. Similarly, when cells were treated with GGTi-298, a significant 31% decrease (*P* = 0.0006) in bacterial viability was observed (Fig. 9C). Concomitantly, ELISA analysis of this supernatant showed that IL-1β production increased by 363.69% (*P* < 0.0001) in these conditions, reinforcing this correlation (Fig. 9D). These data reinforce the hypothesis that part of the effect of statins on the intracellular viability of *M. leprae* comes from its impact on IL-1β production via decreased protein prenylation.

## DISCUSSION

The MP, responsible for the biosynthesis of cholesterol and isoprenoids, plays a key role in leprosy pathogenesis (25). LDs, enriched in cholesterol esters, are induced by *M. leprae* in infected macrophages through increased cholesterol synthesis and uptake, and inhibition of MP with statins accelerates *M. leprae* killing, both *in vitro* and *in vivo* (25, 26). In a recent study, our group showed that *M. leprae* can oxidize host cell cholesterol to cholestenone, generating reductive power for ATP and mycobacterial lipid synthesis (28). Despite the known relevance of MP in *M. leprae* survival, its full significance remains unclear since the potential role of isoprenoids, also major products of MP, in *M. leprae*–macrophage interaction has never been explored. Previous studies have demonstrated a connection between the activity of prenylated GTPases and regulation of immune response (8–10, 45). Herein, we investigated the possibility that the blockage of isoprenoid synthesis would also be contributing to bacterial killing in macrophages treated with statins. This is the first study to demonstrate the involvement of the isoprenoids in the interaction between *M. leprae* and the host cell, advancing our understanding of the MP involvement in leprosy.

Irradiated *M. leprae* retains the same immunogenic and metabolic-modulating properties, including the ability to induce lipid droplet formation in host macrophages (36). This makes it a valuable and safe tool for studying the early stages of *M. leprae–*host interaction, especially to immunometabolism and innate immune signaling. Gamma irradiation does not significantly alter the bacillus’ surface structures, lipids, or pathogen-associated molecular patterns (PAMPs), which are crucial for host immune recognition (46). A significant limitation in working with live *M. leprae* is its inability to be cultured *in vitro*, necessitating propagation in animal models such as the mouse footpad or armadillo (47). These methods are logistically demanding and costly and yield limited quantities of viable bacteria. As a result, irradiated *M. leprae* represents a practical and biologically relevant alternative for *in vitro* studies focused on host–pathogen interactions, especially those investigating innate immune responses and metabolic reprogramming in macrophages.

Our findings indicate that *M. leprae*-stimulated macrophages behave similarly to non-stimulated cells when MP is inhibited. Cells treated with MP inhibitors showed increased IL-1β production, and this effect was reversed by GGPP administration, demonstrating the importance of protein geranylgeranylation in regulating IL-1β production. Additionally, the geranylgeranyl transferase-1 inhibitor, GGTi-298, significantly increased IL-1β and IL-18 production in macrophages, implying the involvement of GTPases prenylated by GGTase-1 in this effect. We also showed that the increased IL-1β and IL-18 production occurred through inflammasome assembly and caspase-1 activation. As previously mentioned, infection with *M. leprae* results in elevated intracellular cholesterol levels in host cells, and this effect is reversed through treatment with statins. The influence of amino bisphosphonates, such as pamidronate, on cholesterol synthesis has also been documented in another model. In contrast, GGTI-298 inhibits an enzyme outside the central axis of the MP that leads to cholesterol synthesis, but does not directly interfere with cholesterol production. Nevertheless, studies indicate that the accumulation of GGPP, the substrate for GGTase-1, can inhibit HMG-CoA reductase activity through feedback regulation. This inhibition may lead to a decrease in cholesterol synthesis. To rule out the possibility that the observed increase in IL-1β following GGTase-1 inhibition is related to decreased cholesterol levels, we investigated whether supplementation with lanosterol—a precursor in the cholesterol biosynthetic pathway—could restore IL-1β levels in GGTI-298-treated cells. Our findings show that lanosterol did not increase IL-1β production nor reverse the effects of GGTI-298, suggesting that the modulation of IL-1β in our system is not due to cholesterol depletion. Instead, it reinforces the idea that results from interference with other branches of the MP, particularly the prenylation of small GTPases. Pamidronate and atorvastatin treatment showed no effect on IL-6 and IL-8 production, suggesting a lack of direct influence of protein prenylation on the production of these inflammatory cytokines in *M. leprae-*stimulated THP-1 macrophages.

We also analyzed the potential association among inhibition of isoprenoid synthesis/protein prenylation, IL-1β production, and intracellular *M. leprae* viability. Analysis of supernatants from infected THP-1 macrophages treated with pamidronate or GGTi-298 showed a significant upregulation of IL-1β production and subsequent decrease with GGPP addition, in a similar way to cells treated with dead bacteria. Of note, IL-1β production inversely correlated with bacterial viability. A slightly lower effect on the decrease in *M. leprae* viability in the condition treated with GGTi-298, compared to the condition treated with pamidronate, might be explained by the fact that pamidronate also inhibits cholesterol synthesis by inhibiting FPPS, and that cholesterol has been shown to be essential for lipid droplets accumulation and *M. leprae* viability (25, 26, 28). On the other hand, GGTi-298 is a specific inhibitor for GGTase-1, the transferase responsible for prenylating geranylgeranylated proteins that do not affect cholesterol biosynthesis. Altogether, these results demonstrate that the upregulation of IL-1β correlates with the decrease in *M. leprae* viability, and that this effect is, at least partially, linked to the isoprenoid GGPP and protein geranylgeranylation catalyzed by GGTase-1.

Interestingly, a significantly higher impact of MP inhibitors was observed in *M. leprae-*stimulated cells when compared to non-stimulated cells. This is likely due to the recognition of bacterial pathogen-associated molecular patterns (PAMPs), which provide a stronger first signal for inflammasome activation compared to PMA treatment alone, which also provides the first signal for inflammasome activation, as demonstrated in the literature (48). Subsequently, impaired prenylation would function as the secondary signal, likewise observed in MKD patients (10, 11). A well-established link exists between reduced geranylgeranylation of GTPases like Rac1 and RhoA and enhanced IL-1β production. This involves an upregulation in the activity of these proteins, leading to the activation of caspase-1 and culminating in the release of IL-1β and IL-18 (10, 11). The upregulation of the activity of Rac1 GTPase is caused by a higher affinity of Rac1 for its respective activating protein (Iqgap1), which has been shown to be caused by a reduction in the prenylation of this GTPase, resulting in a higher GTP load and consequently higher activity of Rac1 (9).

Besides its influence on the innate immune response, the blockage of protein prenylation may potentially affect other cellular processes relevant in the context of *M. leprae*–macrophage interaction. One example is the formation of LDs, previously shown to be induced by *M. leprae-*infected macrophages (25). For instance, the proteins RalA and Rab18, small GTPases that contain geranylgeranyl motifs and are prenylated by GGTase-1 (49, 50), play pivotal roles in the regulation of LD dynamics, particularly in coordinating LD formation, trafficking, and interactions with other organelles (51, 52). For example, Rab18 has been linked to lipid metabolism and LD biogenesis, with the geranylgeranylation modification required for initial endoplasmic reticulum membrane association and contact with LDs, which increases the size of newly formed LDs and facilitates the transfer of cargo to these organelles (51). RalA has also been shown to be associated with the biogenesis and distribution of LDs within the cell (52). Thus, RalA and Rab18 coordinate LDs’ spatial organization and functional regulation, ensuring their integration into broader cellular metabolic and signaling networks. Thus, regulating LD dynamics by protein prenylation is a relevant topic but goes beyond this study and needs further exploration.

In this manner, further studies are being performed to explore the effects of geranylgeranylation on LD biogenesis during *M. leprae* infection. In addition, protein prenylation has also emerged as a critical post-translational modification in pathogens such as *Salmonella typhimurium*, *M. tuberculosis*, and *Legionella pneumophila*. The literature has shown that these pathogens exploit host cell prenyltransferases and isoprenoids to modify their proteins, significantly enhancing their ability to survive within host environments (53–55). Further studies are being conducted to try and determine if *M. leprae* is acting also in an equivalent way, using host machinery for prenylating their own proteins, through the analysis of the presence of the “CAAX” signal peptide for prenylation.

Based on the observations exposed above, we hypothesize that the upregulation of the MP by *M. leprae* in infected macrophages contributes to bacterial survival and persistence in the host in at least two different ways: (i) by increasing the levels of intracellular cholesterol used by the bacteria for reductive power generation, as previously shown (28); and (ii) by increasing the rates of protein prenylation, including proteins of the small GTPases superfamily, which downmodulates the inflammasome activation and subsequent generation of the pro-inflammatory cytokines IL-1β and IL-18. Further research is required to fully understand the role of protein prenylation, especially geranylgeranylation, in leprosy.

In conclusion, protein prenylation have emerged as a novel area of study, with potential applications in controlling infections, including antiparasitic, respiratory syncytial virus, *Salmonella enterica* serovar Typhimurium, and in antitumor activity, where GGTi-298 has shown therapeutic potential (56–61). Faced with the need for novel therapeutic strategies for leprosy, our findings point to protein prenylation as a promising new drug target to control the disease. Moving in this direction, prenylome analysis through LC-MS/MS is underway to identify the specific proteins undergoing prenylation, as well as their prenylation status in *M. leprae-*infected cells. This will allow for further investigation of these proteins, with the ultimate goal of identifying novel therapeutic targets. Furthermore, molecular docking studies are also underway to identify specific inhibitors of geranyl transferases, aiming to reduce *M. leprae* viability while minimizing side effects for patients.

## SUPPLEMENTAL MATERIAL

Figure S1 jb.00185-25-s0001.pdf Evaluation of the effects of pamidronate and GGPP on cell viability.

Figure S2 jb.00185-25-s0002.pdf Evaluation of GGTi-298 effects on cell viability.

ASM does not own the copyrights to Supplemental Material that may be linked to, or accessed through, an article. The authors have granted ASM a non-exclusive, world-wide license to publish the Supplemental Material files. Please contact the corresponding author directly for reuse.
