# Supplementary material for: Impact of protein prenylation inhibition on Mycobacterium leprae viability and IL-1β production in infected macrophages
Source: J Bacteriol. 2025 Aug 27;207(9):e00185-25. doi: 10.1128/jb.00185-25 (PMC12445085; doi:10.1128/jb.00185-25)
Supplement: Figure S1 — Evaluation of the effects of pamidronate and GGPP on cell viability. [file jb.00185-25-s0001.pdf]

SUPPLEMENTARY FIGURE 1

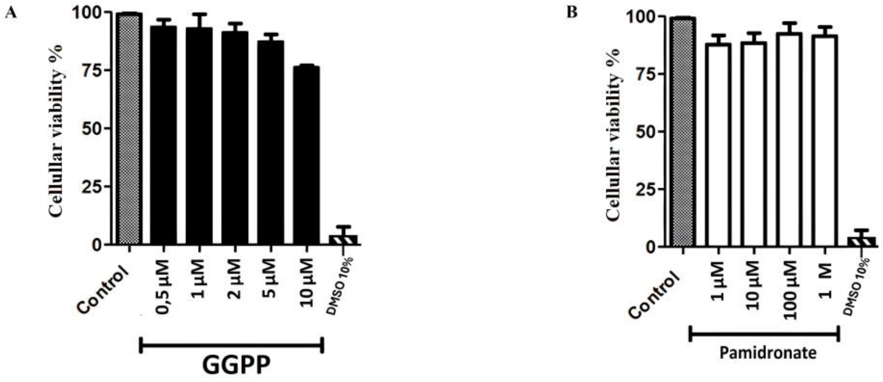

Supplementary Figure 1: Evaluation of the effects of Pamidronate and GGPP on the cell viability: THP-1 macrophages were stimulated or not with (A) GGPP (0,5µM, 1µM, 2µM, 5µM and 10µM) or DMSO (10%) used as a death positive control, and (B) pamidronate (1µM, 10µM, 100µM and 1M) for 24 hours, at 37ºC and 5%CO_2. The cellular viability was assessed by MTT assay. All data are mean ± SD.
