# Supplementary material for: Impact of protein prenylation inhibition on Mycobacterium leprae viability and IL-1β production in infected macrophages
Source: J Bacteriol. 2025 Aug 27;207(9):e00185-25. doi: 10.1128/jb.00185-25 (PMC12445085; doi:10.1128/jb.00185-25)
Supplement: Figure S2 — Evaluation of GGTi-298 effects on cell viability. [file jb.00185-25-s0002.pdf]

SUPPLEMENTARY FIGURE 2

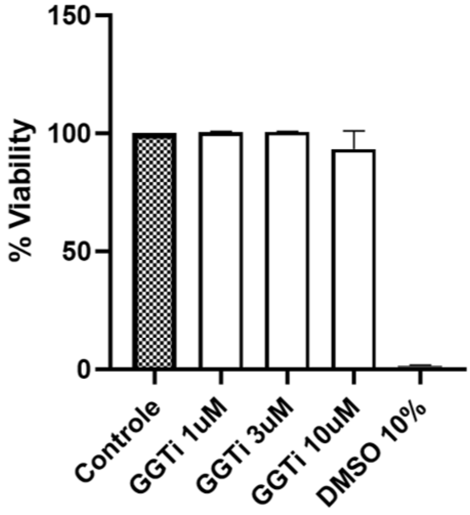

Supplementary Figure 2: Evaluation of GGTi-298 Effects on Cell Viability: THP-1 macrophages were stimulated or not with GGTi-298 (1µM, 3µM and 10µM) or DMSO (10%) used as a death positive control for 24 hours, at 37ºC and 5%CO 2 to evaluate cellular viability by MTT assay. All data are mean ± SD.
